# Dopamine-beta hydroxylase polymorphism and cocaine addiction

**DOI:** 10.1186/1744-9081-4-1

**Published:** 2008-01-03

**Authors:** Camila Guindalini, Ronaldo Laranjeira, David Collier, Guilherme Messas, Homero Vallada, Gerome Breen

**Affiliations:** 1 MRC Social Genetic and Developmental Psychiatry Research Centre, Institute of Psychiatry, King's College London, UK; 2 Laboratório Integrado de Neurociências Clínicas, Department of Psychiatry, Federal University of São Paulo, Brazil; 3 Department of Psychobiology, Federal University of São Paulo, Brazil; 4 Department of Psychiatry, University of São Paulo, Medical School, Brazil; 5 UNIAD (Unit of Drug and Alcohol Research), Department of Psychiatry, Federal University of São Paulo, Brazil; 6 Division of Psychological Medicine, Institute of Psychiatry, King's College London, UK

## Abstract

Cocaine addiction involves a number of medical, psychological and social problems. Understanding the genetic aetiology of this disorder will be essential for design of effective treatments. Dopamine-beta hydroxylase (DbH) catalyzes the conversion of dopamine to norepinephrine and could, therefore, have an influence on both cocaine action and the basal sensitivity of neurotransmitter systems to cocaine. Recently, the -1021C>T polymorphism have been found to strongly correlated with individual variation in plasma DbH activity. To test the influence of this polymorphism on the susceptibility of cocaine addiction, we decided to genotype it in a sample of 689 cocaine addicts and 832 healthy individuals. Genotypic and allelic analyses did not show any evidence of association with cocaine addiction, even after correcting for the effect of population stratification and other possible confounders. Our results do not support a major role of the -1021C>T polymorphism or the gene itself in the development of cocaine addiction but further examination of other variants within this gene will be necessary to completely rule out an effect.

## Findings

Cocaine is one of the most powerfully addictive of the drugs of abuse. The number of cocaine users is estimated at some 13 million worldwide [1]. From those, 15–16% will become addicted within 10 years of first cocaine use [2]. Twin and family studies have demonstrated that cocaine addiction has a strong genetic component but the exact basis of the heritable factors that have a significant contribution to this phenotype remain unclear [3].

Cocaine's potent actions in blocking the uptake by neuronal plasma membrane transporters for dopamine (DAT), serotonin (SERT), and norepinephrine (NET) are well known [4]. Studies in transgenic mice indicate that both DAT and SERT can mediate cocaine's rewarding effects, but the DAT may play the more important role [5]. On the other hand, mice lacking norepinephrine transporter demonstrated prolonged clearance of NE, elevated extracellular levels of this catecholamine and were behaviourally hypersensitive to cocaine and amphetamine, as measured by locomotor stimulation and conditioned place preference [6]. Similarly, double knockouts of both SERT and NET showed dramatically enhanced cocaine place preference [7].

Dopamine-beta hydroxylase (DbH) catalyzes the conversion of dopamine to norepinephrine (NE) and could, therefore, have an influence on both cocaine action and the basal sensitivity of neurotransmitter systems to cocaine [8]. It has been demonstrated that DbH knockout mice are hypersensitive to the psychomotor, rewarding, and aversive effects of cocaine, as measured by locomotor activity and conditioned place preference [9]. Pharmacological treatment studies with the DbH inhibitor disulfiram also indicate that this medication has efficacy as a treatment for cocaine dependence [10,11].

DbH plasma activity levels are reported to vary widely among individuals [12]. Several studies have attempted to find genetic polymorphisms related to differences in enzyme levels. [13-15]. Cubells and colleagues found that a 19 base pair insertion/deletion polymorphism and the SNP 444A>G were associated with plasma DbH levels and that alleles of similar association to enzymatic levels were in significant positive disequilibrium. Additionally, the haplotype containing the two low DbH activity alleles was also associated with cocaine-induced paranoia [15].

More recently, using extreme phenotypes in samples from African and European American and Japanese populations, Zabetian and co-workers [16] reported that the -1021C>T polymorphism (rs1611115) located in the 5' flanking region of the gene accounted for between 35–52% of the variation in plasma DbH activity in these populations. More strikingly, individuals with CC genotype had on average eleven times the enzyme activity of individuals carrying TT genotype [16]. Most recently, a study has shown that cocaine users homozygous for the T allele have an increased propensity to paranoia over time during cocaine self-administration [17].

However, no study has specifically tested the role of this variation in cocaine addiction aetiology, where it may be expected that individuals with the CC genotype might be more resistant to cocaine's effects. To verify the influence of this polymorphism on the susceptibility to cocaine addiction, we decided to conduct an association study in our previously described sample [18,19] of 689 cocaine addicts and 832 healthy individuals, from Sao Paulo, Brazil.

The genotyping of the -1021C>T polymorphism was performed under contract by K-Biosciences (Cambridge, UK) blind to status using an amplifluor assay. Hardy-Weinberg equilibrium, genotype and allele frequencies were compared using a χ^2 ^test and odds ratios and 95% confidence intervals were derived from logistic regression, using SPSS v12.0. We selected a total of 71 (64 SNPs and 7 microsatellites) ancestry-informative markers, e.g. markers that exhibit large allele frequency differences among the three main Brazilian ancestral populations (Europeans, Africans and Native American) to correct for potential population stratification in the sample, using the program ADMIXMAP [20]. The list of markers can be obtained upon request.

The genotype frequencies were in Hardy-Weinberg equilibrium in both, case and control groups for the -1021C>T polymorphism. The genotyping failure rate was around 3%. Genotype counts and frequencies of the polymorphism in both groups, as well as allele wise odds ratios analyses, can be found in Table 1. Genotypic and allelic distribution did not provide any evidence for association between the marker and cocaine addiction (χ^2 ^= 1.01; df = 2; p = 0.59 and χ^2 ^= 0.83; df = 1; p = 0.91; OR = 1.08; 95%CI = 0.91–1.28). The correction for population stratification performed by ADMIXMAP did not affect the allelic association test (z = -0.626; two-tailed p value = 0.53). Further genotypic analyses, dividing the sample by sex, did not provide evidence of association for males (χ^2 ^= 0.83; df = 2; p = 0.66) and females (χ^2 ^= 0.19; df = 2; p = 0.91).

**Table 1:** Genotype and allele counts and frequencies (%) of the -1021C>T polymorphism (rs1611115) in the DbH gene among healthy controls and cocaine addicts.

**Genotype-Wise**									**Allele-Wise**					
	**CC**		**CT**		**TT**		**Total**	**pvalue**	**C**		**T**		**pvalue**	**OR (95%IC)**

Controls	487	59%	294	35%	51	6%	832	0.59	1268	76%	396	24%	0.35	1.08(0.9–1.28)
Cases	421	61%	228	33%	40	6%	689		1070	78%	308	22%		

Finally, evaluation of different models considering a dominant and a recessive effect for the low activity allele (T) also did not show difference between cases and controls, after correction for the potential confounding variables sex, age and education (OR = 0.91; 95%CI = 0.72–1.15; p > 0.05) and (OR = 1.07; 95%CI = 0.66–1.74; p > 0.05).

The neurotransmitter norepinephrine and the norepinephrine transporter play an important role in the rewarding effects of cocaine [6,7], suggesting that genetic variation in the genes involved in the noradrenergic transmission could be responsible for differences in the individual response to cocaine. The DbH gene was an interesting candidate to test this hypothesis since it encodes for the enzyme responsible for the production of NE and consequently for the control of the NE/DA ratio in noradrenergic neurons. The strong association reported between -1021C>T genotypes and DbH levels, robustly indicated that this, or another polymorphism in very tight LD, might be controlling the variation in enzymatic levels and NE synthesis observed across individuals [8] and could, therefore, account for increased susceptibility to abuse cocaine.

However, genotypic and allelic distribution, as well as the evaluation of recessive or dominant models for the low activity variant in a Brazilian sample of 689 cocaine addicts and 832 healthy controls did not provide evidence of association between this variant and the trait under study, even after correction for sex age, education and population stratification.

This is the first study examining the effect of polymorphisms in the DbH gene and the susceptibility to cocaine addiction utilizing a case-control approach. Cubells and co-workers (2000) [15] have studied cocaine dependent subjects and demonstrated an association with the low activity alleles of the insertion/deletion polymorphism and the SNP 444A>G, but with the development of cocaine induced paranoia. The results of our study corroborate with the findings by Köhnke et al. (2002) [21] and Cubells (2002) [22]. Both groups demonstrated that plasma DbH activity was significantly lower in alcoholic subjects and in individuals with unipolar major depression with psychotic features, respectively. However, these positive associations were independent of genotype at -1021C>T, e.g. genotypic and allelic distribution for this polymorphism did not significantly differ between the groups under study. More recent studies also failed to find an association between this polymorphism and epilepsy [23], schizophrenia [24] and Tourette Syndrome [25].

In summary, our results do not support a specific role for the -1021C>T in cocaine addiction in the Brazilian population or a major role for variation in enzymatic activity of DbH. However, our study does not exclude a minor role for the DbH protein or its related pathway in the development of cocaine addiction and suggests that the examination of other variants within this gene not in close LD with the -1021C>T is necessary to completely rule out an effect.

## Competing interests

The author(s) declare that they have no competing interests.

## Authors' contributions

CG and GB analyzed the data, carried out statistical analysis and wrote the paper. RL was the psychiatrist coordinator of the sample collection. RL, DC and GM and HV participated in study design, and helped to revise drafts of the manuscript. All authors read and approved the final manuscript.
